# Depth-of-Field-Extended Plenoptic Camera Based on Tunable Multi-Focus Liquid-Crystal Microlens Array

**DOI:** 10.3390/s20154142

**Published:** 2020-07-25

**Authors:** Mingce Chen, Wenda He, Dong Wei, Chai Hu, Jiashuo Shi, Xinyu Zhang, Haiwei Wang, Changsheng Xie

**Affiliations:** 1 National Key Laboratory of Science & Technology on Multispectral Information Processing, Huazhong University of Science & Technology, Wuhan 430074, China; D201780651@hust.edu.cn (M.C.); M201872714@hust.edu.cn (W.H.); D201677599@hust.edu.cn (D.W.); D201880681@hust.edu.cn (C.H.); D201980727@hust.edu.cn (J.S.); 2 School of Artificial Intelligence and Automation, Huazhong University of Science & Technology, Wuhan 430074, China; 3 Innovation Institute, Huazhong University of Science & Technology, Wuhan 430074, China; 4 Wuhan National Laboratory for Optoelectronics, Huazhong University of Science & Technology, Wuhan 430074, China; hiway@hust.edu.cn (H.W.); Cs_xie@hust.edu.cn (C.X.)

**Keywords:** liquid-crystal (LC) device, plenoptic camera, depth-of-field (DOF) extension, 2D/3D switchable function

## Abstract

Plenoptic cameras have received a wide range of research interest because it can record the 4D plenoptic function or radiance including the radiation power and ray direction. One of its important applications is digital refocusing, which can obtain 2D images focused at different depths. To achieve digital refocusing in a wide range, a large depth of field (DOF) is needed, but there are fundamental optical limitations to this. In this paper, we proposed a plenoptic camera with an extended DOF by integrating a main lens, a tunable multi-focus liquid-crystal microlens array (TMF-LCMLA), and a complementary metal oxide semiconductor (CMOS) sensor together. The TMF-LCMLA was fabricated by traditional photolithography and standard microelectronic techniques, and its optical characteristics including interference patterns, focal lengths, and point spread functions (PSFs) were experimentally analyzed. Experiments demonstrated that the proposed plenoptic camera has a wider range of digital refocusing compared to the plenoptic camera based on a conventional liquid-crystal microlens array (LCMLA) with only one corresponding focal length at a certain voltage, which is equivalent to the extension of DOF. In addition, it also has a 2D/3D switchable function, which is not available with conventional plenoptic cameras.

## 1. Introduction

Conventional cameras only record 2D light intensity information, not most of the information about the light distribution. Unlike conventional cameras, plenoptic cameras can record the 3D information of objects (i.e., the 4D plenoptic function or radiance, also called the light-field) including the radiation power and the ray direction. The capture of high-dimensional data sets that contain rich scene information enable many unique applications of plenoptic cameras, such as digital refocusing [1,2], multi-view imaging [3,4], depth estimation [5,6], and 3D reconstruction [7,8,9]. The first prototype of the plenoptic camera was proposed by Adelson and Wang in 1992 [10]. Then, Ng et al. simplified the design and built the first hand-held plenoptic camera based on the conventional photography camera in 2005, which is called the standard plenoptic camera [1]. In 2009, Lumsdaine and Georgiev proposed a focused plenoptic camera, which is also called plenoptic camera 2.0. Compared to the standard plenoptic camera, the focused plenoptic camera can achieve a much higher spatial resolution of the rendered images but still with a limited depth of field (DOF). Nowadays, there are three kinds of plenoptic cameras that attract a wide range of research interest: A plenoptic camera based on a microlens array (MLA) [1,2,3,4,11,12,13], a plenoptic camera based on a camera array [14], and a plenoptic camera based on a coded mask [15]. Specifically, among the three main designs, the plenoptic camera based on the MLA has a very promising development prospect due to its small size, low cost, and light-field acquisition in a single photographic exposure. Recently, liquid-crystal microlens array (LCMLA)-based plenoptic cameras have attracted a lot of research interest [16,17,18,19], which is owed to the advantages of easy integration with other devices, 2D/3D switchability, ease of manufacture [18,19,20,21,22,23], and, most important, excellent tunability due to the redistribution of liquid-crystal (LC) molecules in the applied electric field [24,25,26,27,28,29,30,31,32,33,34,35,36,37]. Such an excellent tunability enables 2D/3D switchable modes and an extended DOF for LCMLA-based plenoptic cameras [17,18,30].

Unlike the traditional mechanical focusing method, digital refocusing relies entirely on digital calculation. By projecting the acquired 4D light-field to different image planes for integral superposition, the in-focus images of different image planes can be obtained. DOF is an important factor that directly affects the range of digital refocusing; a larger DOF corresponds to a wider range of digital refocusing. To achieve a wide range of digital refocusing, multi-focus plenoptic cameras have been proposed, some of which use an array of interleaved microlenses with different focal lengths, while others use three interlaced hexagonal microlens arrays with different lens types [38,39], focused at two or more different planes. Although a wider range of digital refocusing is obtained, the focal length of each microlens is fixed, which limits its application to some extent. In LCMLA-based applications, DOF extension can be acquired via patterned electrodes of multi-regions and multi-apertures [21,40]; the former obtains multi-focus function by applying different voltages at different regions, while the latter utilizes interleaved apertures to obtain multi-focus function of the entire region with only one voltage. Both electrode designs provide effective approaches to further extend the range of digital refocusing, i.e., the DOF of plenoptic cameras.

In this paper, we demonstrate a plenoptic camera based on the tunable multi-focus (TMF)-LCMLA for achieving a larger DOF. The proposed plenoptic camera consists of a conventional main lens, a TMF-LCMLA, and a complementary metal oxide semiconductor (CMOS) sensor. The specific electrode design of the TMF-LCMLA produces a multi-focus function with only one voltage signal, and the multi-focus function is electrically tunable with applied voltage signals. Experiments demonstrated that the proposed plenoptic camera can acquire a much wider range of digital refocusing compared to the plenoptic camera based on a conventional LCMLA with only one corresponding focal length at a certain voltage, which is equivalent to the extension of DOF. In addition, it also has 2D/3D switchable functionality, which is not available with conventional plenoptic cameras.

## 2. Materials and Methods

### 2.1. Structure of TMF-LCMLA

**Figure 1:**
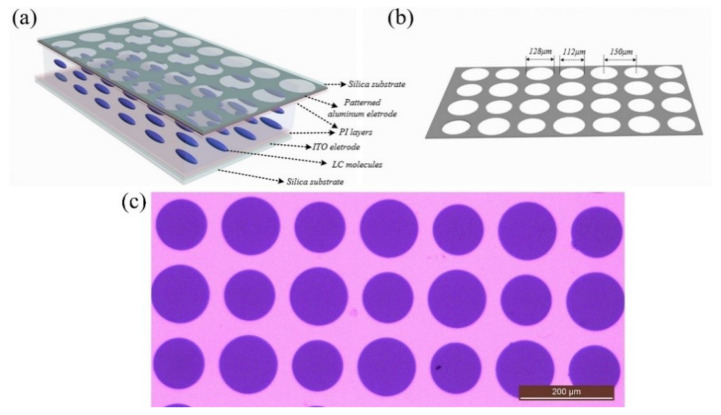
(**a**) The schematic of the tunable multi-focus liquid-crystal microlens array (TMF-LCMLA), (**b**) structural parameters of micro-holes on the patterned aluminum electrode, and (**c**) microscopic picture of micro-holes on the patterned aluminum electrode.

As a key component of the proposed plenoptic camera, the schematic of the TMF-LCMLA is depicted in Figure 1a. The key functional structures of the TMF-LCMLA are both ~500 μm silica substrates with different conductive films pre-coated over their inner surface. The inner surface of the bottom substrate is deposited with a planar indium tin oxide (ITO) electrode of 185 nm and the inner surface of the top substrate is deposited with an aluminum electrode of 100 nm. The method used is magnetron sputtering. After conventional ultraviolet photolithography and the wet-etching process, the patterned aluminum electrode with interleaved micro-holes of two different diameters is formed. The structural parameters and microscopic picture of micro-holes on patterned aluminum are shown in Figure 1b,c, respectively. As shown, the diameters of small and large micro-holes are 112 and 128 μm, respectively, and the center-to-center distance is 150 μm. Polyimide (PI) layers (ZKPI-440 of POME Technology Co., LTD., Beijing, China) are continuously spin-coated on the patterned aluminum and planar ITO electrodes of the TMF-LCMLA, and then prebaked for 10 min at 80 °C and cured for 30 min at 230 °C, sequentially. Both prebake and cure operations are performed at the hot plate. The cured PI layers on the patterned aluminum and planar ITO electrodes act as an alignment layer and are rubbed anti-parallel to each other for homogeneously aligning the LC molecules filled later. Glass microsphere spacers of 25 μm diameter, mixed with the adhesive, are deposited to separate the two substrates. Finally, a layer of long rod-shaped nematic LC materials (E44 of Merck) is filled in the formed micro-cavity. The electro-optical parameters are: ne = 1.7904 and no = 1.5277 (∆*n* = 0.2627), and ε_⊥_ = 5.2, ε_//_ = 22.0, where ε_⊥_ and ε_//_ are the dielectric constants of the LC molecules perpendicular or parallel to the director, respectively.

### 2.2. Principle of TMF-LCMLA

**Figure 2:**
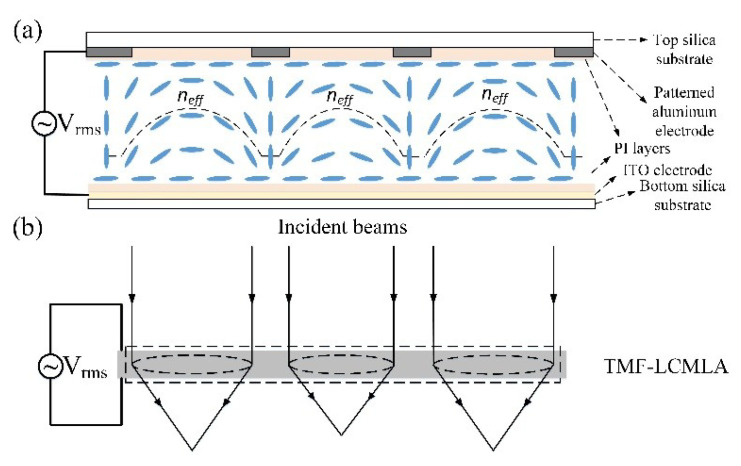
A detailed schematic of the TMF-LCMLA used in the plenoptic camera proposed by us. (**a**) Gradient refractive index profiles of extraordinary ray in TMF-LCMLA when a driving voltage signal in an appropriate range is applied, and (**b**) the lens-like effect of TMF-LCMLA, noting that the focal lengths of the liquid-crystal (LC) microlens with different apertures are different.

Due to the birefringence characteristics of a nematic LC, an incident light ray is split into two components (the ordinary ray and extraordinary ray) whose polarization directions are orthogonal to each other when it enters. The refractive index of the ordinary ray is independent of the orientation of the incident ray entering. On the contrary, the refractive index of the extraordinary ray is dependent on the angle between the incident ray and the orientation of the optical axis of nematic LC materials, which depends on the LC director orientation [41]. Therefore, when the LC director is tilted, supposing the orientation of the incident ray is fixed, a variation in the refractive index of the extraordinary ray is formed, forming a lens-like effect. For the TMF-LCMLA, the tilting is attained by use of an electric field. A detailed schematic of the TMF-LCMLA used in the plenoptic camera proposed by us is given in Figure 2.

As Figure 2a shows, when a driving voltage signal in an appropriate range is applied on the TMF-LCMLA, gradient refractive index profiles of the extraordinary ray are formed due to the redistribution of liquid-crystal molecules, where the gradient refractive index profiles corresponding to the micro-holes of different apertures are different, so they have different focal lengths. Compared to micro-holes of small aperture, micro-holes of large aperture have a flatter refractive index profile, resulting in a larger focal length. The black dashed line in the LC cell indicates the equivalent refractive index profile of the extraordinary ray. Figure 2b demonstrates the lens-like effect of the TMF-LCMLA. When the TMF-LCMLA is driven by an appropriate voltage signal, incident beams with a specific polarization direction (i.e., parallel to the rubbing direction of PI layers) can be converged to different planes corresponding to LC microlenses of different apertures. Thus, the TMF-LCMLA acts as a multifocal array with only one voltage control; note that the focal lengths can be adjusted by tuning the root-mean-square (RMS) value of the driving voltage signal applied.

### 2.3. Principle of Plenoptic Camera Based on TMF-LCMLA

**Figure 3:**
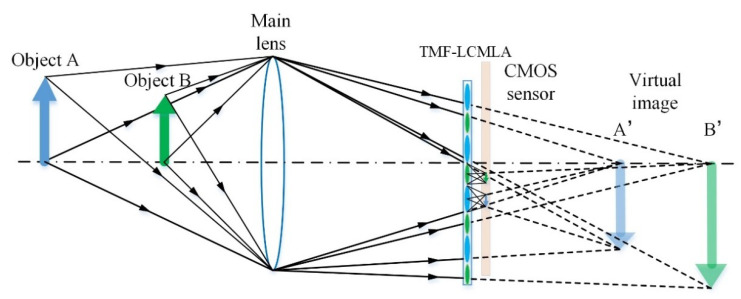
The schematic of the plenoptic camera based on TMF-LCMLA working in Galilean mode.

The schematic of the proposed plenoptic camera is shown in Figure 3. The main lens and TMF-LCMLA constitute two imaging subsystems. First, incident light from objects is compressed by the main lens, where the main lens tends to form images A’ and B’, which are behind the CMOS sensor without considering the TMF-LCMLA, so the images A’ and B’ can be treated as virtual, in which case they are known as the Galilean mode [42]. Secondly, virtual images are projected by LC microlenses as objectives and, finally, an array of elemental images is formed on the CMOS sensor. In this case, a perfectly focused system satisfies the lens equation:

(1) $$-\frac{1}{a}+\frac{1}{b}=\frac{1}{f}.$$

where *a* represents the distance from the TMF-LCMLA to the virtual image, *b* represents the distance from the TMF-LCMLA to the CMOS sensor, and *f* is the focal length of the microlens. When the mathematical relationship between *a*, *b*, and *f* deviates from the lens equation, the image becomes blurred, which is the fundamental optical limitations to the range of digital refocusing. Thanks to the tunable multifocal function of the TMF-LCMLA, clear images of objects in a much wider depth range can be acquired with only one voltage signal. As Figure 3 shows, with an appropriate voltage signal, clear images of both objects A and B can be acquired by LC microlenses with large aperture and small aperture, respectively. In addition, limited by the lens equation, they are unable to clearly image another object, which means a much wider range of digital refocusing (i.e., a larger DOF) compared to the plenoptic camera based on the conventional LCMLA with only one corresponding focal length at a certain voltage. It should be noted that the image of the main lens may be a real one if it is formed in front of the image plane, which is known as the Keplerian mode. Similarly, a much wider range of digital refocusing can also be obtained with only one applied voltage signal, which means DOF extension as well.

### 2.4. Digital Refocusing Range

As described in the previous section, diffraction is an optical effect that limits the total resolution limit of the proposed plenoptic camera. In its presence, a point object is not imaged as a point, but spreads into a finite-size image spot. For an ideal circular aperture, the 2D diffraction pattern is called the Airy disk. According to the Rayleigh criterion, the separation *s_λ_* of two adjacent Airy disks should at least be equal to the distance from the center of the Airy disk to the first dark ring for distinguishing adjacent Airy disks. Generally, *s_λ_* is approximately given by:

(2) $$s_{\lambda }=1.22\lambda (f/\#)$$

where *λ* represents the wavelength and *f*/# denotes the *F*-number of the imaging system. Another important factor that affects the resolution limit is the pixel pitch *P_p_* and, thus, *s* determines the resolution limit, which is defined as:

(3) $$s=\max[s_{\lambda },p_{p}]$$

**Figure 4:**
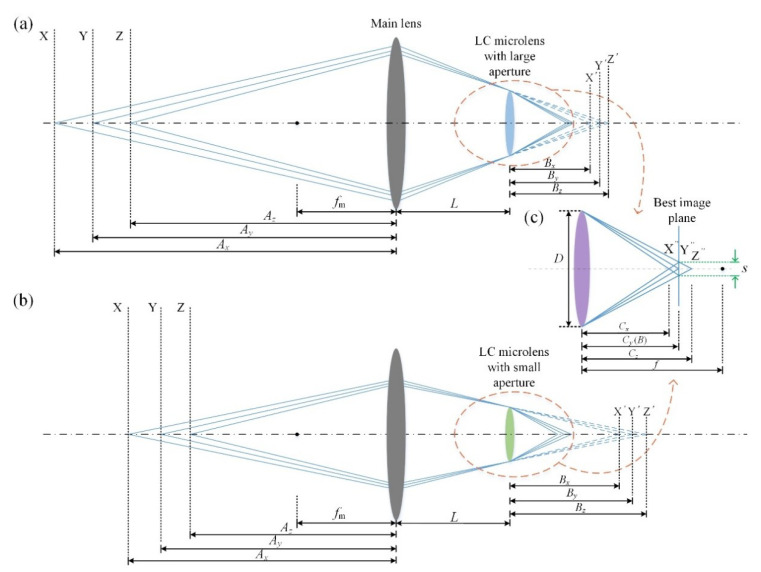
Digital refocusing range of the proposed plenoptic camera (**a**) corresponding to the case of the LC microlens with large aperture, and (**b**) corresponding to the case of the LC microlens with small aperture. (**c**) The schematic diagram of imaging of LC microlens.

Figure 4 shows a schematic of the digital refocusing range of the proposed plenoptic camera. *L* presents the distance from the main lens to the LC microlens, *B* presents the distance from the LC microlens to the sensor plane, and *f_m_* is the focal length of the main lens. As shown in Figure 4a,b, lights from points X, Y, and Z are compressed by the main lens and tend to form points X’, Y’, and Z’. However, due to the presence of the LC microlens, they are eventually imaged at the points X”, Y”, and Z”, which is shown in Figure 4c. *C_x_*, *C_y_*, and *C_z_* are the distances from points X”, Y”, and Z” to the LC microlens. Point Y” is just on the best image plane (i.e., the sensor plane) and, thus, *C_y_* is equal to *B*. X” and Z” are two points before and behind the best image plane, respectively, and the spot sizes are exactly equal to *s*. In this case, they are considered to be acceptably sharp. According to previous studies, the mathematical relationships are as follows [18]:

(4) $$\left\{ \begin{array}{l} A_{x}={\left(\frac{1}{f_{m}}-\frac{1}{B_{x}+L}\right)}^{-1},A_{z}={\left(\frac{1}{f_{m}}-\frac{1}{B_{z}+L}\right)}^{-1}\\ B_{x}={\left(\frac{1}{f}-\frac{1}{C_{x}}\right)}^{-1},B_{z}={\left(\frac{1}{f}-\frac{1}{C_{z}}\right)}^{-1}\\ C_{x}=\frac{BD}{D+s},C_{z}=\frac{BD}{D-s} \end{array}\right.$$

where *B_x_* and *B_z_* are the distances from the LC microlens to the points X’ and Z’. *A_x_* and *A_z_* are the distances from the main lens to the points X and Z, and *f_m_* represents the focal length of the main lens. 

Therefore, the digital refocusing range can be considered as between *A_x_* and *A_z_* in theory. At a certain voltage, considering the case of the LC microlens with different apertures (i.e., 112 and 128 μm), the changes in the fundamental variables are *D* and *f_m_*. According to Equation (4), *A_x_* and *A_z_* will also be different with this change, which means the digital refocusing range of different apertures is different, indicating a wider digital refocusing range, as shown in Figure 4. The DOF extension depends on the mathematical relationship between *A_x_* and *A_z_* corresponding to different-aperture LC microlenses. The maximum DOF extension can be achieved when the digital refocusing ranges corresponding to the LC microlens with different apertures do not coincide at all. The premise of satisfying this situation is that the focal length difference between them cannot be too small. In addition, at this time, compared to the plenoptic camera based on the LCMLA with large aperture, the DOF extension is the DOF of the plenoptic camera based on the LCMLA with small aperture. Compared to the plenoptic camera based on the LCMLA with small aperture, the DOF extension is the DOF of the plenoptic camera based on the LCMLA with large aperture.

## 3. Results

### 3.1. Optical Features of TMF-LCMLA

**Figure 5:**
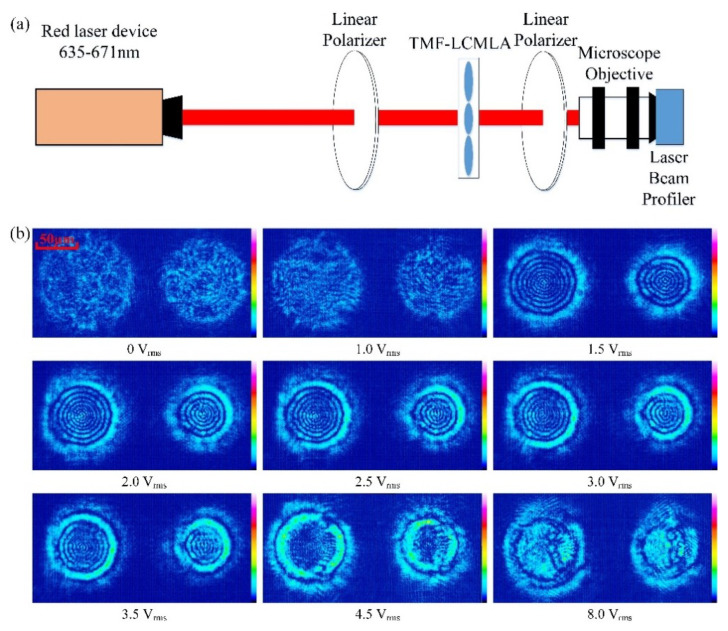
(**a**) The optical system for measuring interference patterns; the rubbing direction of the TMF-LCMLA is oriented at 45° with respect to the transmission axis of the two crossed polarizers. (**b**) The measured interference patterns with different applied voltage signals of 1 kHz.

To evaluate the performance of the TMF-LCMLA, interference patterns are first measured. The measuring system is demonstrated in Figure 5a. A beam of 635–671 nm from a red laser device (Changchun New Industries Optoelectronics Tech. Co., Ltd., Changchun, China) passes through the TMF-LCMLA cell placed between two crossed polarizers. The rubbing direction of the TMF-LCMLA is oriented at 45° with respect to the transmission axis of the two linear polarizers. Subsequently, interference occurs and the interference patterns are captured by a laser beam profiler (WinCamD of DataRay, Inc., Redding, CA, USA) with a microscope objective of ×40 and 0.65 numerical aperture. The retardation difference of the two adjacent constructive or destructive interference rings indicates a phase change of 2π. 

Figure 5b shows the measured interference patterns with different applied voltage signals of 1 kHz. For LC microlenses with large aperture, the maximum phase difference is Δδ~9 × 2π, i.e., nine rings, which is very close to the theoretical value calculated by the following formula [43]:

(5) $$\delta =2\pi d_{LC}\Delta n/\lambda$$

where *d_LC_* represents the thickness of the LC layer, 25 μm, ∆*n* represents birefringence, 0.2627, and *λ* represents the wavelength of the laser, 635–671 nm, and the calculated maximum phase difference is thus ~20.69π. 

According to the results, interference patterns can be well formed with an applied voltage signal of 1.5–3.0 V_rms_, which is the appropriate voltage range for the TMF-LCMLA to have lens-like effects. The intervals between every two fringes are not much different, indicating that the index is distributed with a smooth gradient, resulting in good lens quality [44].

**Figure 6:**
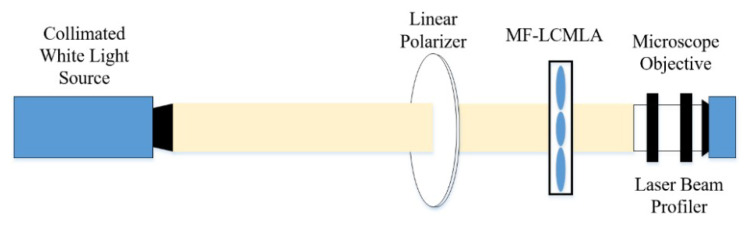
The testing system for measuring the relatively accurate value of the focal length of the TMF-LCMLA. The transmission axis of the linear polarizer is parallel to the rubbing direction of the polyimide (PI) layers.

For the TMF-LCMLA, the focal length is an important parameter. The approximate focal length value can be calculated based on the measured interference patterns, as shown below [31]:

(6) $$f=r^{2}/2\lambda N$$

where *r* is the radius of the lens aperture and *N* is the number of observed rings. When a voltage signal is applied, the relatively accurate value of focal length is obtained by measuring the sharpest light intensity distribution [21]. The testing system is demonstrated in Figure 6. A collimated white light source is first polarized by a linear polarizer (USP-50C0.4-38 of OptoSigma, Tokyo, Japan), and then continuously passes through the TMF-LCMLA. The transmission axis of the linear polarizer is parallel to the rubbing direction of the PI layers. Then, the light-fields are remarkably amplified by a microscope objective, and finally captured by a laser beam profiler (WinCamD of DataRay, Inc., Redding, CA, USA). To finely locate the focal planes shaped, we precisely adjust the distance between the TMF-LCMLA and the microscope objective for obtaining the sharpest light intensity distribution of the converged light-fields. The relatively accurate value of focal length is equal to the sum of the thickness of the silica substrate and the distance between the exiting end of the TMF-LCMLA and the incident surface of the microscope.

**Figure 7:**
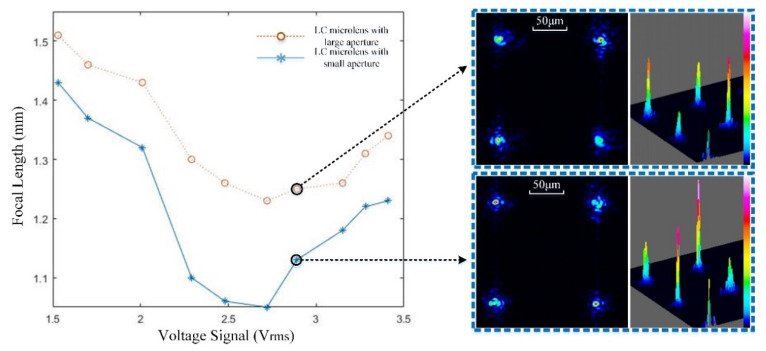
Relationship between the focal length of the TMF-LCMLA and the RMS value of the voltage signals applied. Figures in the dotted boxes on the right indicate the 2D and 3D point spread functions (PSFs) of the TMF-LCMLA with a voltage signal of ~2.89 V_rms_.

Figure 7 demonstrates the relationship between the focal length of the TMF-LCMLA and the RMS value of the voltage signal applied. The voltage signal is an AC square wave with a frequency of 1 kHz. Figures in the dotted box on the right indicate the 2D and 3D point spread functions (PSFs) of the TMF-LCMLA, respectively, with a voltage signal of ~2.89 V_rms_. It is worth mentioning that the LC refractive index cannot be a perfect axisymmetric parabolic-like distribution, and it has dispersion, which results in imaging aberrations such as spherical and chromatic aberrations [45,46]. The actual full-width at half-maxima (FWHMs) of focal spots shown in Figure 7 are between ~2 and ~3 μm. According to our experiments, the current TMF-LCMLA can work normally in the range from ∼1.5 to ∼3.4 V_rms_, and the focal length is in the range from ∼1.05 to ∼1.51 mm, which shows excellent tenability at low voltage and is relatively consistent with the measured interference patterns. With different voltage signals in an appropriate range, the TMF-LCMLA acts as a tunable multifocal array. Moreover, with the same voltage signal applied, the focal length of the LC microlens with large aperture is always larger than that with small aperture, which is consistent with the theory. 

### 3.2. Imaging Application of Plenoptic Camera Based on TMF-LCMLA

**Figure 8:**
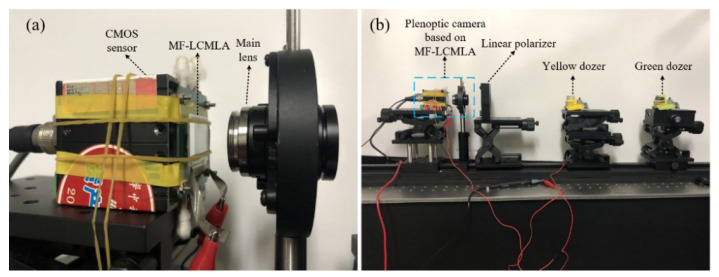
Photographs of: (**a**) The proposed plenoptic camera prototype, and (**b**) the experimental diagram for plenoptic imaging.

The prototype of the proposed plenoptic camera and the experiment diagram of plenoptic imaging are illustrated in Figure 8. As shown in Figure 8a, the proposed plenoptic camera consists of a CMOS sensor, a fabricated TMF-LCMLA, and a main lens (M3520-MPW2 of Computar) with a focal length of 35 mm. The resolution of the CMOS sensor array (MVC14KSAC-GE6 of Microview) is 4384 × 3288 and the pixel pitch is 1.4 μm. The F-number of the main lens is set as 5.6 to match that of the LC microlenses, which can effectively eliminate the crosstalk between sub-images under a large aperture and thus make relatively full use of the resolution of the CMOS sensor.

Figure 8b demonstrates the experiment diagram for plenoptic imaging. The yellow dozer and green dozer were placed 240 and 470 mm away from the proposed plenoptic camera, respectively. The linear polarizer (USP-50C0.4-38 of OptoSigma, Tokyo, Japan) between the two dozers and proposed plenoptic camera is used to satisfy the polarization sensitivity of the TMF-LCMLA, thereby eliminating stray-light crosstalk. The transmission axis of the linear polarizer is parallel to the rubbing direction of the PI layers.

**Figure 9:**
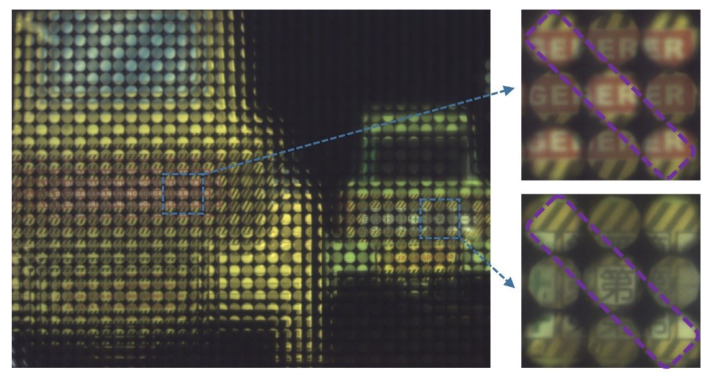
The light-field image and partially enlarged images captured by plenoptic camera based on TMF-LCMLA.

Figure 9 shows the light-field image captured by the plenoptic camera based on the TMF-LCMLA; the applied voltage signal is ~2.0 V_rms_. Partial images of yellow and green dozers are shown on the right side, and microimages in the purple dotted frame are captured by LC microlenses with large aperture. As shown, microimages of the yellow dozer captured by LC microlenses with small aperture (i.e., 112 μm) are in-focus, whereas the LC microlenses with large aperture (i.e., 128 μm) capture the out-of-focus images. Conversely, for the green dozer farther away, microimages captured by LC microlenses with large aperture and small aperture are in focus and indistinguishable, respectively. Notice the differently focused microimages, interleaved.

**Figure 10:**
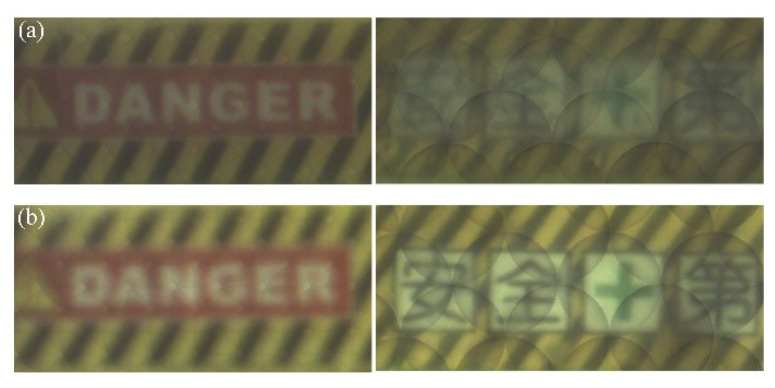
Partial rendering images from microimages captured by (**a**) LC microlenses with small aperture, and (**b**) LC microlenses with large aperture.

Digital refocusing is performed based on the raw light-field image in Figure 9 to obtain rendering images of different image planes. The partial rendering images from microimages captured by LC microlenses with small aperture and large aperture are shown in Figure 10a,b, respectively. As shown, the refocused yellow dozer (i.e., the dozer with ‘danger’) by LC microlenses with small aperture is in-focus while the same object refocused by LC microlenses with large aperture is out-of-focus. The refocused green dozer (i.e., the dozer with Chinese characters) by LC microlenses with large aperture is very sharp while the same object refocused by LC microlenses with small aperture is in low contrast, which is perceived as blurrier [47]. The relationship of the rendering images is consistent with the captured microimages. It can be found that the two refocused images of the yellow dozer (i.e., the dozer with ‘danger’) are relatively smoother, while there are obvious overlapping shadow noises in the two refocused images of the green dozer (i.e., the dozer with Chinese characters), which is caused by the larger rendering radius.

**Figure 11:**
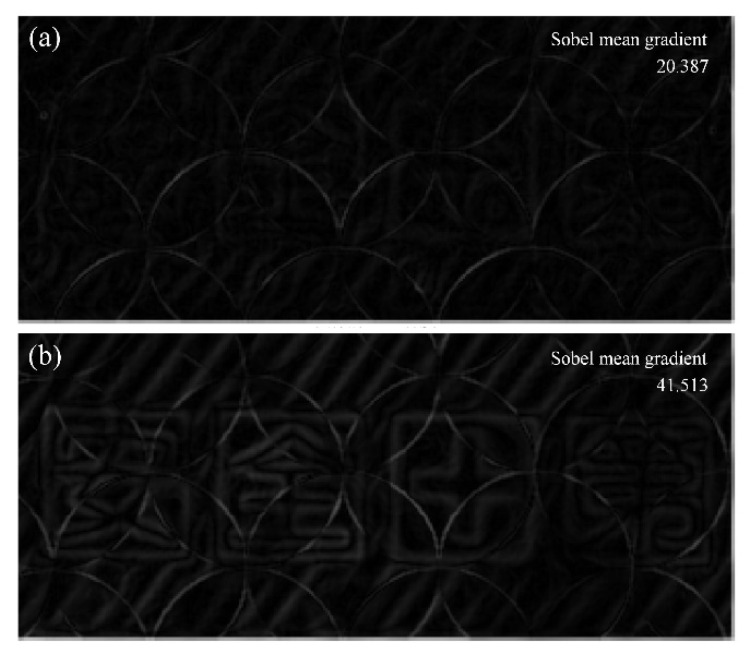
Edge images obtained after the operation of Sobel operator. (**a**) LC microlenses with small aperture, and (**b**) LC microlenses with large aperture.

For these two sets of images with different noise characteristics, different methods are used to objectively and quantitatively evaluate the image quality, thereby demonstrating a wider range of digital refocusing. An image quality evaluation algorithm named BISHARP is used to evaluate the two refocused images of the yellow dozer. The BISHARP algorithm is a fast no-reference image sharpness/blurriness assessment model that operates in the spatial and transform domain [47]. It generates local contrast image maps by computing the root-mean-squared values for each image pixel within a defined local neighborhood size, thus estimating the sharpness of the image. The lower the score of the input image, the sharper it is. Scores of two refocused images of the yellow dozer in Figure 10 are 1.917 and 2.632 corresponding to LC microlenses with small apertures and large apertures. This means that the refocused yellow car by LC microlenses with small aperture is sharper, which is consistent with objective theory and subjective observation. Due to obvious overlapping shadow noises, Sobel edge detection is selected to detect the edge information of the two refocused green dozer images [21]. The Sobel operator is a first-order discrete difference operator that can not only obtain a relatively good detection effect, but also has a smooth suppression effect on noise. It contains two 3 × 3 matrices, which are used to perform the plane convolution operation with the measured image, to obtain the approximate brightness difference in both horizontal and vertical directions. Generally, the sharper image has a faster gray variation at the image edge. The edge images obtained after the operation of the Sobel operator are shown in Figure 11. In Figure 11a, apart from noise, almost no texture features of Chinese characters and lines can be seen, and the texture features of Chinese characters and lines can be clearly seen in Figure 11b. Sobel mean gradients of Figure 11a,b are 20.387 and 41.513, respectively, which means that the refocused green dozer by LC microlenses with large aperture is relatively sharper [21]. Similarly, it is consistent with objective theory and subjective observation.

Therefore, the plenoptic camera based on the TMF-LCMLA has a much wider range of digital refocusing compared to the plenoptic camera based on the conventional LCMLA with only one corresponding focal length at a certain voltage, which is equivalent to the extension of the DOF. It should be noted that the raw light-field image acquired by the proposed plenoptic camera is polarized due to the use of a linear polarizer. Thus, the digital refocusing images are also polarized, and the angle of the polarization is tunable [19], which can effectively introduce polarization information into the plenoptic cameras.

**Figure 12:**
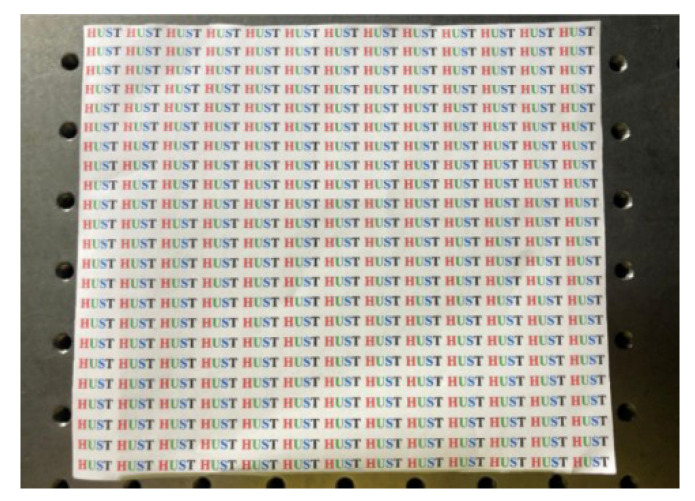
The target used for demonstrating the electrical shift in digital refocusing range.

**Figure 13:**
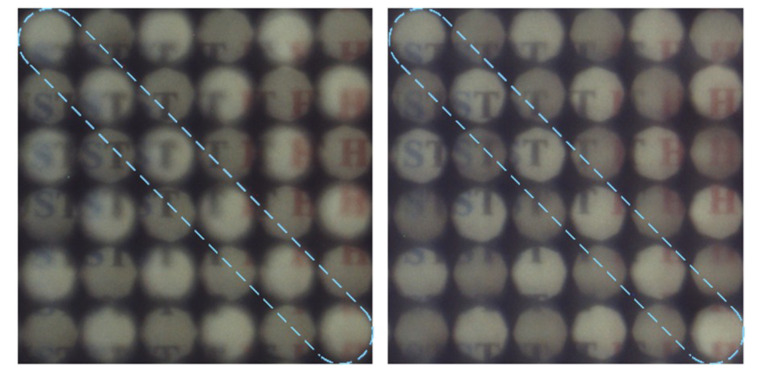
The partial captured light-field images of the object at a distance of 200 mm with applied voltage signals of ~2.20 V_rms_ (**left**) and ~2.75 V_rms_ (**right**). Note the sharpness of the microimages within the blue dotted line.

Moreover, due to the excellent tunability of the TMF-LCMLA, the digital refocusing range of the proposed plenoptic camera can be shifted electrically. A target is shown in Figure 12. On the left and right sides of Figure 13 are partially captured light-field images of the target at a distance of 200 mm with applied voltage signals of ~2.20 V_rms_ and ~2.75 V_rms_, respectively.

The microimages within the blue dotted line are captured by LC microlenses of 128 μm, which are out-of-focus with an applied voltage signal of ~2.20 V_rms_ and in-focus with an applied voltage signal of ~2.75 V_rms_. It is remarkable that the digital refocusing range of the camera can also be efficiently extended simply by changing the applied voltages.

### 3.3. 2D/3D Switchable Function

**Figure 14:**
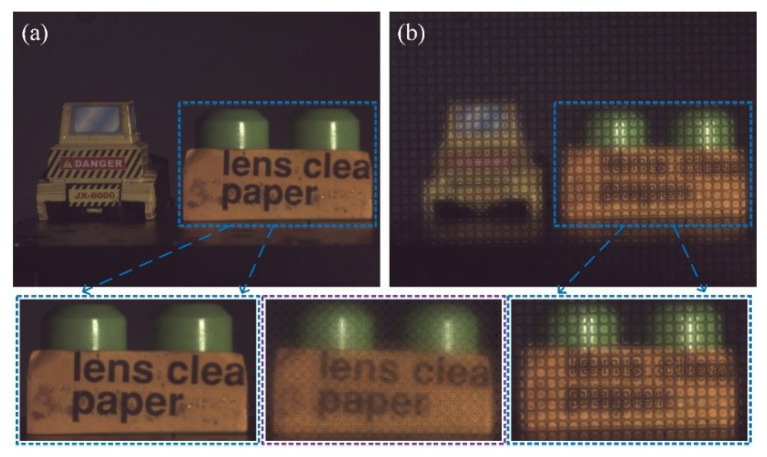
Images obtained in (**a**) full-resolution imaging mode and (**b**) plenoptic imaging mode.

Conventional plenoptic cameras can only work in the plenoptic imaging mode. The raw light-field image obtained in this case is not intuitive, as shown in Figure 9. To obtain a 2D image of the objective world, digital refocusing must be performed. When the material of the patterned electrode is changed from Al to ITO, the full-resolution imaging mode and the plenoptic imaging mode can be respectively obtained with or without applied voltage signals via the proposed plenoptic camera, i.e., the 2D/3D switchable function. Figure 14a,b show the images obtained in full-resolution imaging mode and plenoptic imaging mode, respectively.

In Figure 14a, a driving voltage signal is not applied over the TMF-LCMLA, so it is not in operation, in which case it is equivalent to a phase retarder. As shown, a 2D image can be obtained clearly, just like conventional cameras. In addition, in Figure 14b, a driving voltage signal of 2.50 V_rms_ is applied over the TMF-LCMLA; thus, the proposed plenoptic camera is in plenoptic imaging mode. Images in the blue dashed box below are partially enlarged, and the image in the middle purple dashed box below is a partial image obtained after digital refocusing. It can be seen that compared to the image taken by the conventional camera, the 2D image obtained via digital refocusing has the disadvantages of low spatial resolution and poor contrast, which indicates that the traditional 2D imaging mode still has important value in the field of imaging. Moreover, stray-light crosstalk is introduced due to the high transparency of ITO (compared to Al-patterned electrode), which is also a reason for the contrast reduction.

## 4. Discussion

In summary, we proposed a novel plenoptic camera with extended DOF based on the TMF-LCMLA. The TMF-LCMLA fabricated by us was experimentally analyzed and its key optical characteristics including interference patterns, focal lengths, and PSFs were measured, which maintained good consistency with the theory. Experiments demonstrated that the specific electrode design of the TMF-LCMLA produced a tunable multi-focus function with applied voltage signals, leading to a much wider range of digital refocusing for the proposed plenoptic camera, which is equivalent to the extension of DOF. Moreover, when the material of the patterned electrode is replaced from Al to ITO, the full-resolution imaging mode and the plenoptic imaging mode can be respectively obtained with or without applied voltage signals, i.e., the 2D/3D switchable function. 

Plenoptic cameras based on the TMF-LCMLA demonstrated a promising development prospect, especially in terms of 2D/3D switchability and DOF extension. As the response time of the LC device is reduced, and with optimization of the LC microlens aberration and further study of the polarization-insensitive LC microlens, we believe that it is possible for the TMF-LCMLA to replace traditional solid microlens arrays as an important component of plenoptic cameras in the future.
